# Supplementary figures and images for: Fus depleted oligodendrocytes reduce neuronal damage and attenuate AD progression in the AppNL-G-F mouse
Source: bioRxiv. 2025 Nov 26:2025.11.24.689041. Preprint. [Version 1] doi: 10.1101/2025.11.24.689041 (PMC12697352; doi:10.1101/2025.11.24.689041)

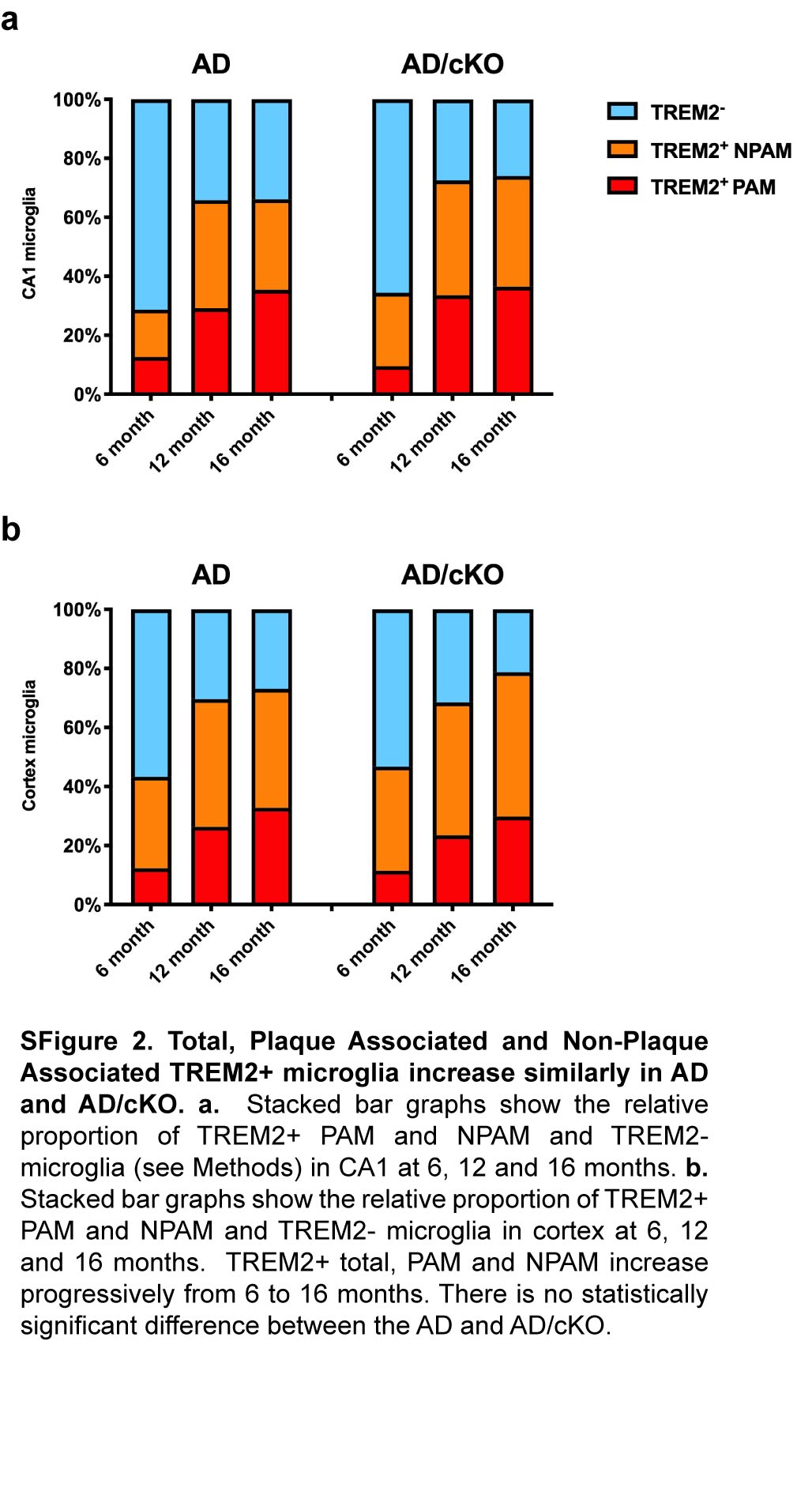

Supplement: Supplement 1 [file media-1.zip › Supplemntary figures_BioRxiv/SFigure 2 -TREM2 PAM.jpg]

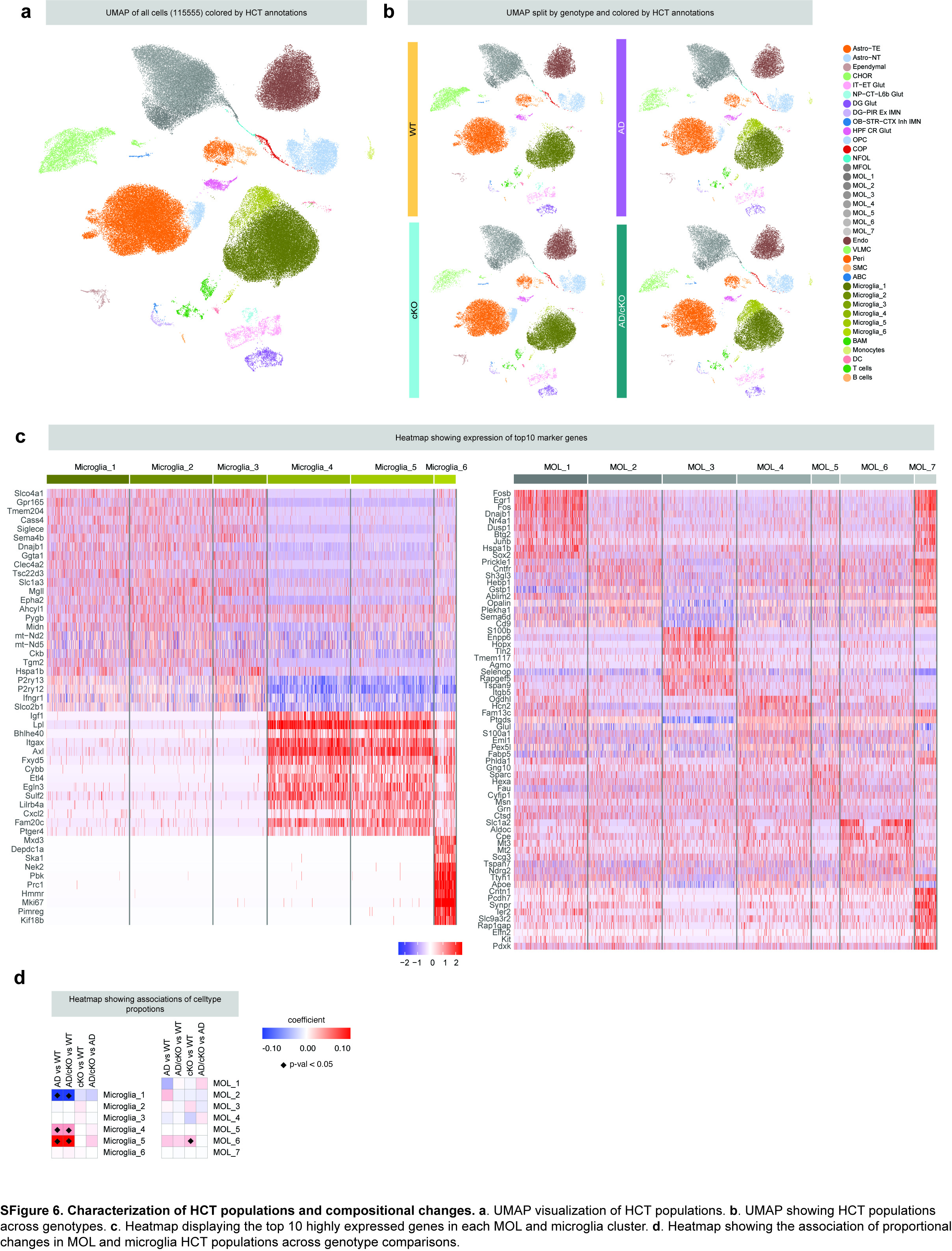

Supplement: Supplement 1 [file media-1.zip › Supplemntary figures_BioRxiv/SFigure 6-SCT3.jpg]

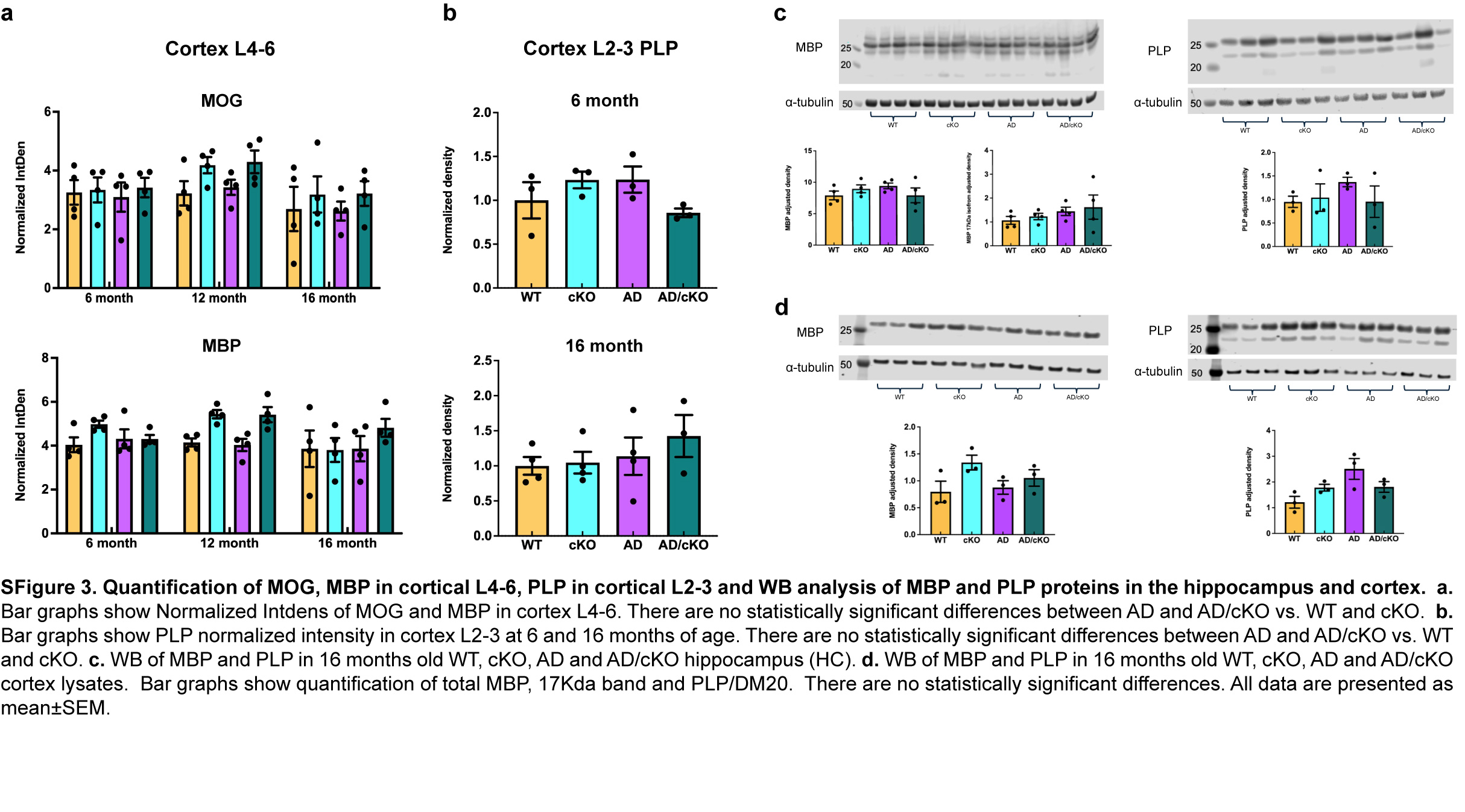

Supplement: Supplement 1 [file media-1.zip › Supplemntary figures_BioRxiv/SFigure 3_L4-6 myelin and WB.jpg]

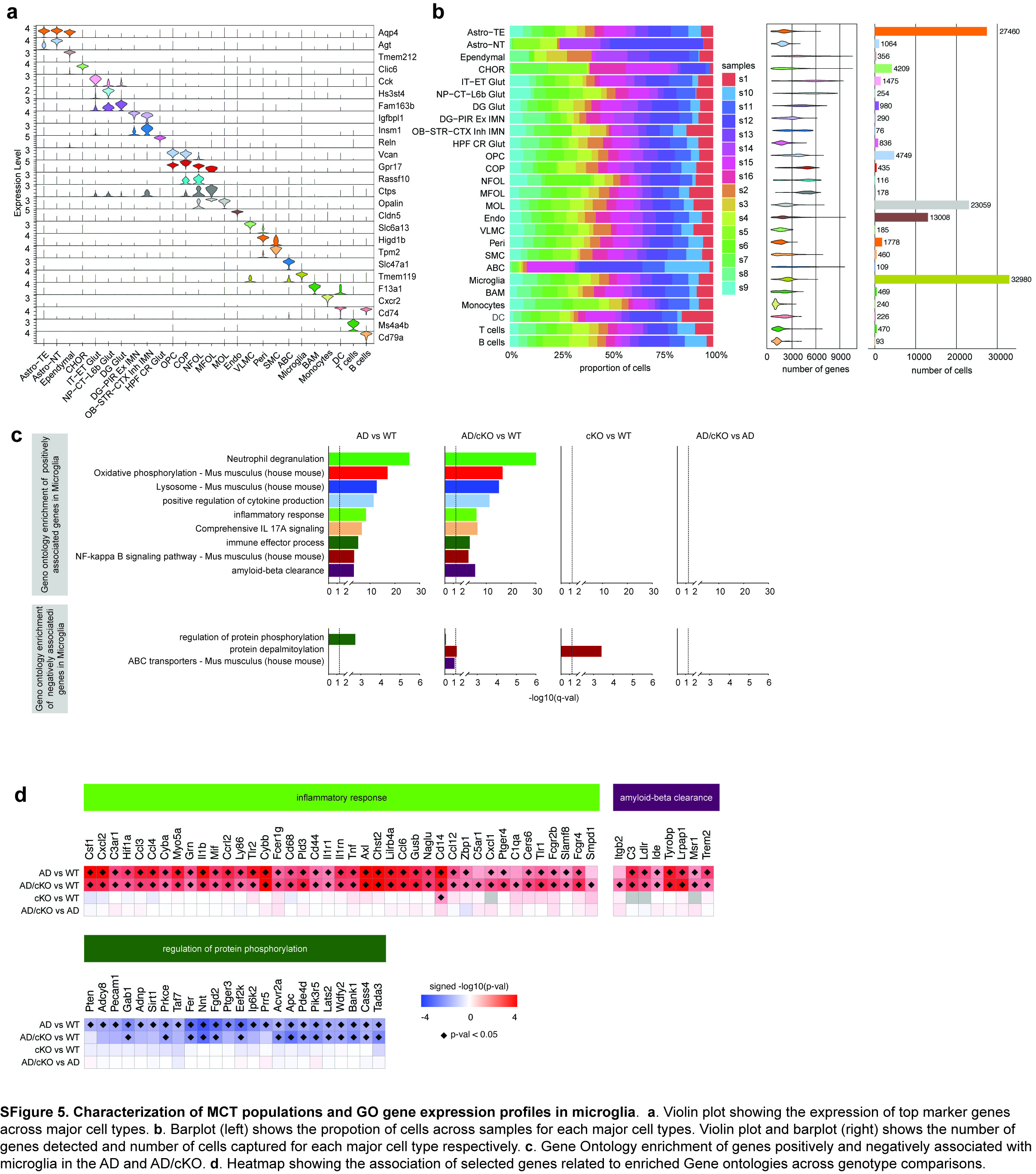

Supplement: Supplement 1 [file media-1.zip › Supplemntary figures_BioRxiv/SFigure 5-SCT2.jpg]

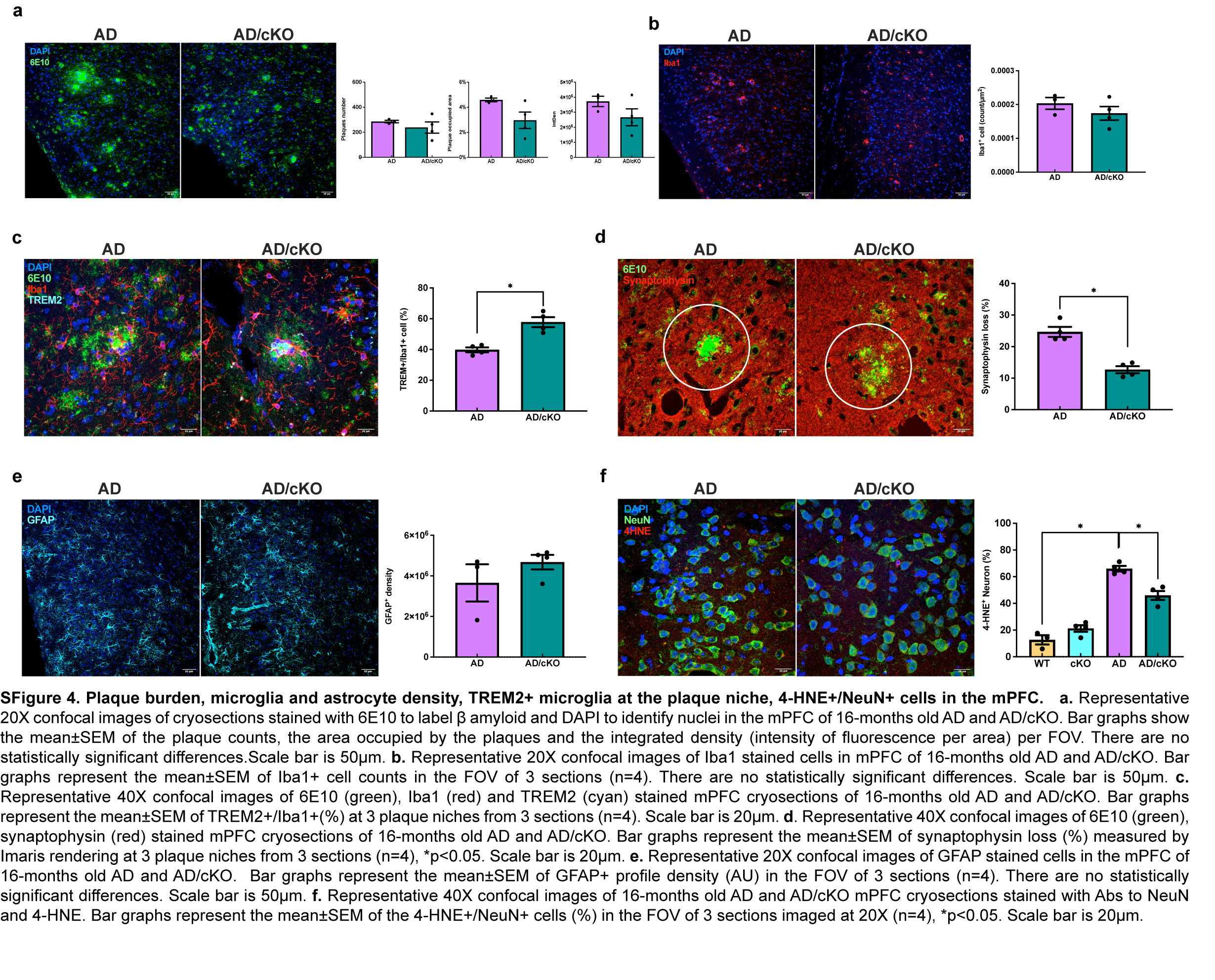

Supplement: Supplement 1 [file media-1.zip › Supplemntary figures_BioRxiv/SFigure 4-mPFC data.jpg]

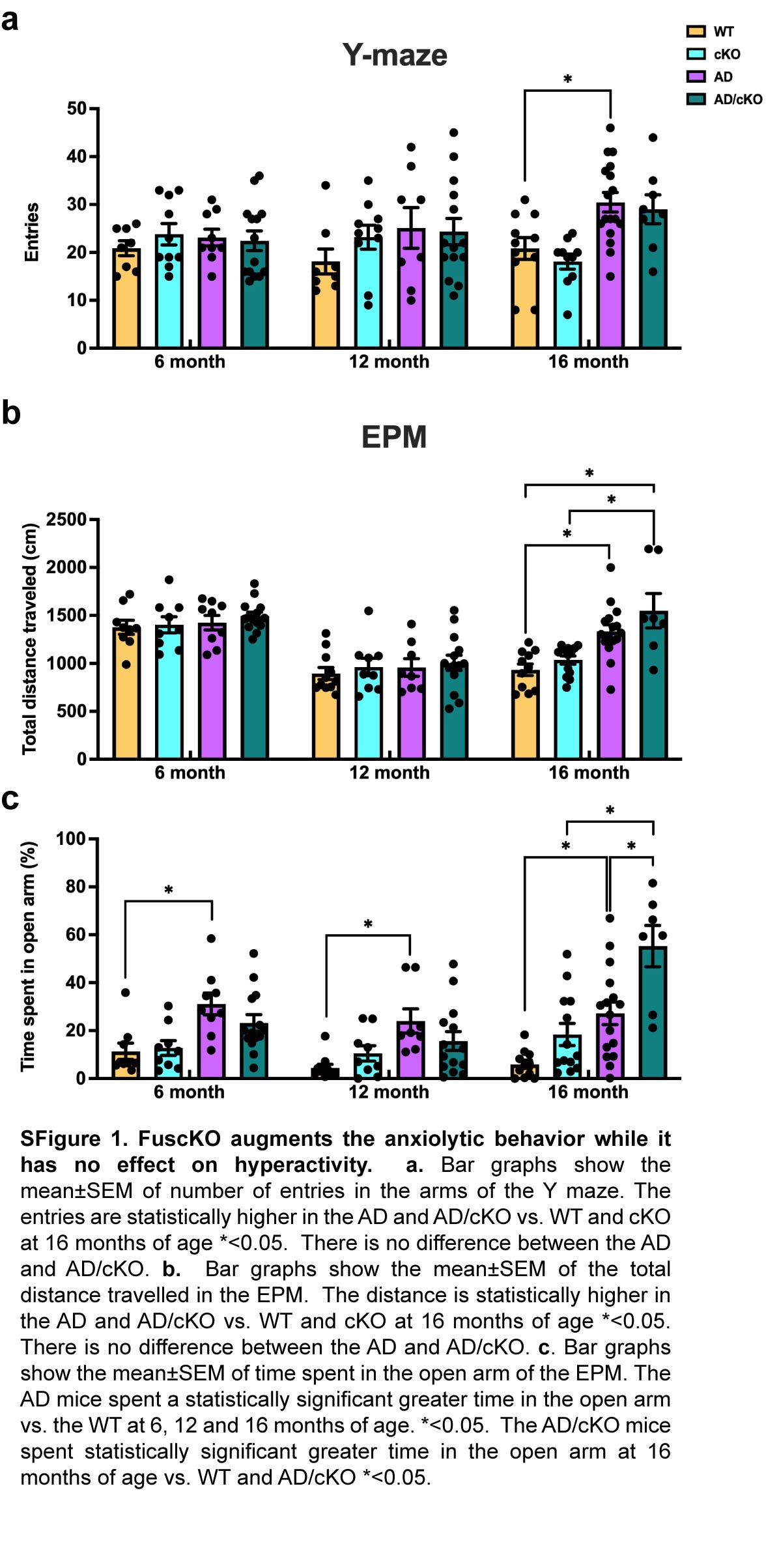

Supplement: Supplement 1 [file media-1.zip › Supplemntary figures_BioRxiv/SFigure 1-anxiety.jpg]
